# Evaluation of New Polyclonal Antibody Developed for Serological Diagnostics of Tomato Mosaic Virus

**DOI:** 10.3390/v14061331

**Published:** 2022-06-18

**Authors:** Michaela Mrkvová, Richard Hančinský, Simona Grešíková, Šarlota Kaňuková, Ján Barilla, Miroslav Glasa, Pavol Hauptvogel, Ján Kraic, Daniel Mihálik

**Affiliations:** 1 Faculty of Natural Sciences, University of Ss. Cyril and Methodius, Nám. J. Herdu 2, 91701 Trnava, Slovakia; michaela.mrkvova@ucm.sk (M.M.); hancinsky1@ucm.sk (R.H.); gresikova1@ucm.sk (S.G.); sarlota.kanukova@ucm.sk (Š.K.); barilla1@ucm.sk (J.B.); miroslav.glasa@ucm.sk (M.G.); daniel.mihalik@ucm.sk (D.M.); 2 National Agricultural and Food Centre, Research Institute of Plant Production, Bratislavská cesta 122, 92168 Piešt’any, Slovakia; pavol.hauptvogel@nppc.sk; 3 Biomedical Research Center of the Slovak Academy of Sciences, Institute of Virology, Dúbravská cesta 9, 84505 Bratislava, Slovakia

**Keywords:** tobamovirus, tomato mosaic virus, tomato, polyclonal antibody

## Abstract

Plant viruses threaten agricultural production by reducing the yield, quality, and economical benefits. Tomato mosaic virus (ToMV) from the genus *Tobamovirus* causes serious losses in the quantity and quality of tomato production. The management of plant protection is very difficult, mainly due to the vector-less transmission of ToMV. Resistant breeding generally has low effectiveness. The most practical approach is the use of a rapid diagnostic assay of the virus’ presence before the symptoms occur in plants, followed by the eradication of virus-infected plants. Such approaches also include serological detection methods (ELISA and Western immunoblotting), where antibodies need to be developed for an immunochemical reaction. The development and characterization of polyclonal antibodies for the detection of ToMV with appropriate parameters (sensitivity, specificity, and cross-reactivity) were the subjects of this study. A new polyclonal antibody, AB-1, was developed in immunized rabbits using the modified oligopeptides with antigenic potential (sequences are revealed) derived from the coat protein of ToMV SL-1. the developed polyclonal antibody. AB-1, showed higher sensitivity when compared with commercially available analogs. It also detected ToMV in infected pepper and eggplant plants, and detected another two tobamoviruses (TMV and PMMoV) and ToMV in soil rhizosphere samples and root residues, even two years after the cultivation of the infected tomato plant.

## 1. Introduction

The tomato (*Solanum lycopersicum* L.) is a member of the *Solanaceae* family that includes more than 3000 plant species. It is an economically important crop cultivated world-wide in almost any climate and environment. Tomato plants constitute a host for many pathogens, including fungi, bacteria, nematodes, and viruses [1,2]. They cause serious economic losses in the quantity and quality of tomato production [3,4]. More than 100 species of plant viruses naturally infect tomato [5,6,7], and they represent a particular risk factor for tomato production systems around the world. An important group of tomato-infecting virus pathogens is the *Tobamovirus* genus, family *Virgaviridae*, including 37 species with a monopartite positive sense ssRNA(+) genome [8].

Many *Tobamoviruses* naturally infect the tomato, such as Tomato mosaic virus (ToMV), Tobacco mild green mosaic virus (TMGMV), Tobacco mosaic virus (TMV), Tomato brown rugose fruit virus (ToBRFV), and Tomato mottle mosaic (ToMMV) [6]. *Tobamoviruses* are considered as unusual plant viruses due to their ability of transmission between host plants without living vectors [9]. *Tobamoviruses* are known as very resilient and remain infectious in contaminated soil and plant debris [10], irrigation water taken from rivers [11], and also in the nutrient solution in hydroponic cultivation systems for a long time [12]. Among them, the Tomato mosaic virus is very harmful, with a wide host range, causing mosaic, stunting, and leaf distortion. It is among the most important viruses of the tomato worldwide. The genome of ToMV consists of at least four open reading frame (ORF)-encoding proteins. Two of them, comprising the replicase components, are encoded by ORF1 (130 kDa) and ORF2 (180 kDa). ORF3 encodes the movement protein (MP) and ORF4 encodes the coat protein (CP) [13]. Besides its function in virion formation, the coat protein of ToMV also plays a role in the long-distance transmission of the virus [14].

There are two main strategies for alleviating the problems of ToMV in tomato growth. One is the genetic resistance of tomato in protecting plants against ToMV. Three dominant resistance genes, *Tm-1*, *Tm-2*, and *Tm-22*, have been found in *Lycopersicon hirsutum* L. and *Lycopersicon peruvianum* L. and were introduced into commercial lines of tomato [15,16]. However, genetic changes through mutation and genetic recombination are generally common for RNA viruses. These lead to the relatively rapid breakdown of ToMV resistance encoded by resistance genes in tomato plants [17,18].

Accurate and sensitive diagnostics of ToMV are the second-most-essential tool for virus disease management in tomato cultivation. Highly specific detection methods are based on the polymerase chain reaction (PCR) and, together with high-throughput sequencing technologies, provide accurate identification of viruses, multiplex virus detection, virus quantification, as well as the opportunity to discover new emerging viruses [19,20]. The high specificity of PCR-based methods can sometimes result in false-negative results as a consequence of nucleotide sequence variation in primer-binding genomic portions occurring in the virus genome by mutation. The presence of PCR inhibitors in analyzed plant samples can also cause problems. Other, newer approaches to the detection of plant viruses are already being used, such as CRISPR/Cas12a-based detection [21] or high-throughput sequencing [22,23]. The latter has brought about an approach for virus identification that does not require knowledge about the virus infecting a given plant. However, the most common methods used for routine plant virus detection remain the enzyme-linked immunosorbent assay (ELISA) and PCR [19,24].

ELISA is usually associated with lower specificity than nucleic acid-based detection [24]. It is also important to distinguish whether the polyclonal or monoclonal antibody is used in the analysis. The use of monoclonal antibodies may not reveal virus strains bearing a mutation in the target universal epitope, as was presented in Plum pox virus detected by the ELISA [25]. Serological methods utilizing polyclonal antibodies provide a versatile tool for the wide screening of plant viruses infecting different crops, although there is a certain risk of a false-positive signal due to the cross-reactivity of polyclonal antibodies with similar viruses [26]. The cross-reactivity is also known from the detection of ToMV using polyclonal antibodies [27]. Manufacturers of commercial ToMV diagnostic ELISA kits and antibodies also usually report cross-reactions with multiple *Tobamoviruses*. Additionally, the International Seed Federation (2019), representing the global seed industry, has drawn attention to the cross-reactivity of polyclonal antibodies for ToMV with other *Tobamoviruses* (TMV, ToMMV, and ToBRFV) [28]. However, due to the wide availability of commercial diagnostic kits and manual simplicity, the ELISA method is probably still the most widely used diagnostic approach in the detection of plant viruses, including ToMV, especially when many samples are being processed. The available commercial polyclonal antibodies for ToMV detection almost always lack detailed information regarding the antigen and epitope used for eliciting an immune response and antibody production. Such information can be obtained in the antibody development process that begins with the isolation of the virus, its characterization, and antigen determination for immunization and antibody production. However, the newly developed antibody must meet similar or better diagnostic properties, a sufficient detection limit, and the desired wider or narrower cross-reactivity in comparison with already available commercial antibodies. The complete process of the preparation of a polyclonal antibody was the subject of this experimental work, assuming that it will be possible to prepare an effective polyclonal antibody. A new polyclonal antibody against ToMV was prepared, and we determined its sensitivity for serological detections of ToMV in tomato plants and related plant species, detected its advantageous cross-reactivity with other *Tobamoviruses*, and tested the antibody for the analysis of the persistence and resilience of ToMV in soil and plant residues.

## 2. Materials and Methods

### 2.1. ToMV SL-1 Isolate and Tomato Genotypes

The ToMV isolate SL-1 was obtained from the collection kept at the Research Institute of Plant Production (Piešťany, Slovakia). The virus originated from a tomato plant manifesting symptoms of virus infection at a local tomato grower in Western Slovakia. Leaves from this plant were collected and stored at −80 °C. The nucleotide sequence of the complete genome of the ToMV SL-1 isolate was determined previously by high-throughput sequencing [29] and included in the GenBank nucleotide database (KY912162, available at: https://www.ncbi.nlm.nih.gov/nucleotide/, accessed on 27 May 2022). This analysis showed that the source tomato plant was infected solely by ToMV without co-infection with other viruses.

The tomato variety Monalbo was used for mechanical artificial inoculation with the original ToMV SL-1 isolate. ToMV SL-1-positive plants were maintained under insect-free conditions, and collected leaves were stored at −80 °C for further experiments. Subsequent artificial infections were conducted in the commercial tomato varieties Mobaci, Monalbo, and Niki Zel F1, and in four breeding lines, 706/15, 730/15, 756/15, and 762/15, developed by Zelseed spol. s r. o. (Senec, Slovakia).

### 2.2. ToMV SL-1 Isolate Characterization

**Figure 1:**
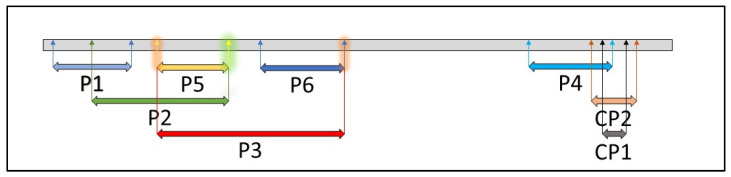
Regions of the ToMV SL-1 genome targeted by primer pairs (Table 1).

Leaf samples from two ToMV SL-1-infected tomato lines (730/15 and 762/15) were used for the confirmation of isolate identity. RNA was isolated using the NucleoSpin RNA Plant Mini kit for RNA from plants (Macherey-Nagel, Düren, Germany) and reversely transcribed to cDNA using the RevertAid First Strand cDNA Synthesis Kit (ThermoFisher Scientific, Waltham, MA, USA). Selected regions of the ToMV genome (Figure 1) were amplified by PCR using eight primer pairs (Table 1) that covered more than two-thirds of the genome, including the coat-protein-coding region. Primers P1–P6 covered a large portion of the nucleotide sequences of both the replication proteins and the movement proteins, as well as almost the entire coat protein. Their positions in the ToMV genome were as follows: 82–949 bp (P1), 527–1888 bp (P2), 1383–3098 bp (P3), 5119–5861 bp (P4), 1383–1882 bp (P5), 2391–3098 bp (P6), 5885–6013 bp (CP1), and 5741–6180 bp (CP2). Amplified fragments were sequenced by the Sanger sequencing method using the Hitachi 3500 Genetic Analyzer (Applied Biosystems, Waltham, MA, USA).

### 2.3. Development ToMV-Specific Polyclonal Antibody

The ORF4 encoding the coat protein of ToMV was selected for antibody development as the most used for antibody development [30].

Specific ToMV epitopes were designed by evaluating the antigenic potential of the amino acid sequences of coat proteins from ToMV SL-1 and fifteen other ToMV isolates retrieved from the UniProt knowledgebase [31] (The UniProt Consortium, 2021, available online: https://www.uniprot.org/uniprot/, accessed on 27 May 2022). Homology in the coat protein sequences within them was in the range of 98.6–99.1% [29]. The nucleotide and amino acid sequences were analyzed using CLC Sequence Viewer 8.0.0 (Qiagen, Aarhus, Denmark) and CLC Main Workbench 8.1 software (Qiagen, Aarhus, Denmark), respectively. Oligopeptide sequences with antigenic potential suitable for generating an immune complex were designed by intersecting desired properties, such as a specific composition of amino acids (cysteine, leucine, and valine) [32] and hydrophobicity [33]. The custom antibody service (Metabion International AG, Planegg, Germany) was used for antibody development. The service included assistance in oligopeptide design, oligopeptide synthesis, animal immunization and development, and immunoaffinity purification. Two amino acid fragments showing the highest coefficient of antigenicity were selected and modified at the N-end or C-end by adding cysteine to increase the immunochemical response of the rabbit. Both oligonucleotides, Epi 1: NH_2_-RFPGDVYKVYRYNAVLDC-COOH and Epi 2: NH_2_-CESMSGLVWTSAPAS-COOH, were used for the immunization of a New Zealand White rabbit by injection with 3 mg of epitope conjugated with keyhole limpet hemocyanin protein (days 1, 21, 28, 35, 42, and 49).

The production of antibody in the rabbit was tested by comparing blood sera before and after immunization on an ELISA reader (Elx800 BioTek, Winooski, VT, USA) at 405 nm wavelength using alkaline phosphatase-conjugated Goat anti-Rabbit IgG (H + L) (Thermo Fisher Scientific, Waltham, MA, USA), whereas immunized sera consistently showed higher absorbance levels, confirming the successful production of polyclonal antibody capable of recognizing ToMV SL-1 coat protein epitopes.

### 2.4. Serological Testing

Tomatoes were mechanically inoculated with ToMV isolate SL-1 for the comparison of the diagnostic properties of the tested antibodies using ELISA. Artificial inoculation was conducted at the stage of two true leaves by deep-frozen ground leaf samples of ToMV SL-1 isolate resuspended in Norit buffer (0.05 M sodium/potassium phosphate buffer, pH 7.0, 1 mM EDTA, 5 mM diethyldithiocarbamic acid, and 5 mM thioglycolic acid; all chemicals were from Merck (KGaA, Darmstadt, Germany). Mechanical wounding with sterile silicon carbide powder Merck(KGaA, Darmstadt, Germany) was used for the inoculation of tomato plants. All plants were cultivated under a photoperiod of 16 h/8 h (light/dark) at 24–25 °C, with light intensity of 152 μmol.m^−2^.s^−1^. The control, non-infected plants were grown under the same conditions. Samples of inoculated plants were collected during the early infection phase (2, 4, 12, and 24 h after inoculation) and also in the late infection phase (7 and 14 days after inoculation).

The sensitivity and specificity of the newly developed polyclonal antibody (AB-1) were tested by a double-antibody sandwich enzyme-linked immunosorbent assay (DAS-ELISA) [34] and Western immunoblotting analysis. Four commercially available antibodies, i.e., ToMV Tomato mosaic virus IgG (art. No. 152615, Bioreba AG, Reinach, Germany), TMV Tobacco mosaic virus IgG (art. No. 190415, Bioreba AG, Reinach, Germany), Tomato mosaic virus antiserum (AS-0104, DMSZ, Braunschweig, Germany), Tomato mosaic tobamovirus Dahlemense, and Positive Control Tomato Mosaic Virus (art. no. 07047S/100 and 07047PC, Loewe Biochemica GmbH, Sauerlach, Germany), were used in the comparison study. The ToMV Tomato mosaic virus conjugate (art. no. 152622, Bioreba AG, Reinach, Germany) was used for conjugation with AB-1 and other primary antibodies, and para-nitrophenylphosphate for the subsequent color reaction measured at 405 nm (Elx800 BioTek, Winooski, VT, USA).

Extracts from fully developed leaves (0.15 g) of the inoculated plant were homogenized in PBS buffer (1/25 *w*/*v*) containing 0.05% Tween-20 and 2% polyvinylpyrrolidone, and subsequently applied to the ELISA plate. All used and compared primary antibodies (commercial and AB-1) were diluted at ratios of 1:100; 1:500; 1:1000; 1:5000; and 1:10,000 (*v*/*v*) starting from the same concentration of 5 μg/μL of total protein. The plant sap was diluted for sensitivity testing at ratios pf 1:10; 1:100; 1:1000; and 1:10,000 (*v*/*v*). Chromogenic reactions were measured by absorbance at 405 nm (Elx800 BioTek, Winooski, VT, USA). All ELISA results that were compared with each other were measured in samples at the same time and on the same ELISA plate.

Proteins for Western immunoblotting analysis were extracted from the tomato plants using the P-PER^®^ Plant Protein Extraction Kit (Thermo Fisher Scientific, Waltham, MA, USA). A standard curve prepared using bovine serum albumin standards was applied for the calculation of the protein concentrations determined using the Pierce ^TM^ BCA Protein Assay Kit (ThermoFisher Scientific, Waltham, MA, USA). The specific protein of interest, representing the ToMV coat protein, was detected by the Western blotting technique [35]. All analyzed samples loaded into the separation gel contained the same amount (50 μg) of total protein. Four ToMV antibodies commercially available at the time of the experiments were used for comparison with the newly developed AB-1 antibody. The expected size of the detected ToMV coat protein of 17.8 kDa in Western blots was determined using the protein molecular weight markers (PageRuler ^TM^ Prestained Protein Ladder 10 to 180 kDa and PageRuler ^TM^ Plus Prestained Protein Ladder 10 to 250 kDa, ThermoFisher Scientific, Waltham, MA, USA). The immunoreactive protein was visualized using the 1-step ^TM^ Ultra TMB-Blotting Substrate Solution (ThermoFisher Scientific, Waltham, MA, USA), recorded using a Nikon D3100 camera (Nikon Corp., Tokyo, Japan), and evaluated using the Image Studio Lite Quantification Software (LI-COR Biosciences, Lincoln, NE, USA).

The cross-reactivity of the AB-1 antibody with TMV and PMMoV viruses was tested in Niki Zel F1 plants mechanically inoculated with five ToMV isolates (PV-0141, PV-0143, PV-0846, PV-1180, PV-0135), one TMV isolate (PV-0107), and one Pepper mild mottle virus (PMMoV, PV-0166). All were obtained from the collection of viruses of the Leibniz Institute DSMZ—German Collection of Microorganisms and Cell Cultures (Braunschweig, Germany).

Systemic infection was tested in pepper (*Capsicum annum* L.) and eggplant (*Solanum melongena* L.). Host plants with 3–4 developed leaves were inoculated with the ToMV SL-1 isolate and analyzed by Western immunoblotting using the AB-1 antibody 21 days after inoculation.

The persistence of ToMV SL-1 was evaluated by analysis of the root residues and rhizosphere soil material. Both were collected from the soil two years after the removal of the ToMV-infected tomato plant using Western blot analysis. The resilience of the ToMV was analyzed in the same samples sterilized at 120 °C for 1, 2, 5, 10, and 20 min.

### 2.5. Statistical Evaluation

The two-way ANOVA and Tukey honest significant difference tests were performed using the software R version 4.0.4 (Free Software Foundation, Boston, MA, USA, available at: https://www.npackd.org/p/r/4.0.4, accessed on 27 May 2022).

## 3. Results

### 3.1. Identity of ToMV SL-1 Isolate

RNA isolated from leaf samples of infected tomato plants 762/15 and 730/15 was used for the confirmation of the identity of ToMV SL-1. ToMV SL-1, used for the inoculation of these plants, was compared with the original nucleotide sequence of ToMV SL-1 isolate [29]. After reverse transcription, the cDNA was amplified using primers specific for ToMV SL-1 coat-protein-coding sequences (CP1 and CP2) and primer pairs P1–P6. The obtained amplicons had the expected size and the Sanger sequencing analysis revealed 100% sequence homology with the previously sequenced ToMV SL-1 isolate [29] in both plants.

### 3.2. Sensitivity of AB-1 Antibody

**Figure 2:**
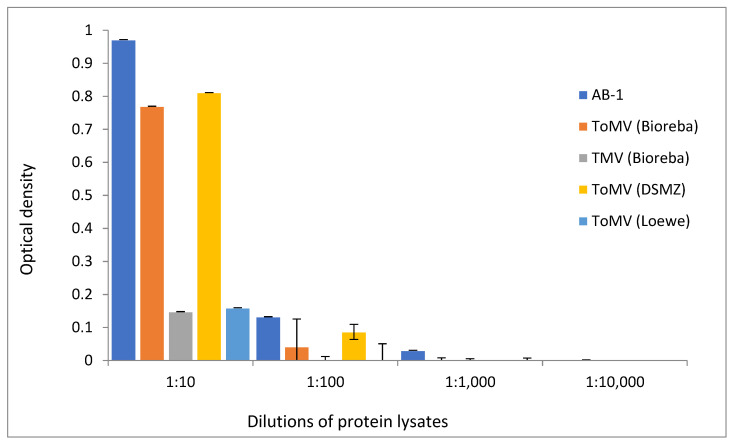
Sensitivity of the compared antibodies in the detection of ToMV by the ELISA assay in differently diluted protein lysates from ToMV-infected tomato plants (mean values and standard deviation error bars).

Proteins extracted from plants of the Niki Zel F1 variety inoculated with ToMV SL-1 were diluted for ELISA analysis at ratios of 1:10; 1:100; 1:1000; and 1:10,000. The presence of ToMV in plant sap was detected using the developed polyclonal antibody AB-1 as well as with three commercially available ToMV antibodies and one TMV antibody. AB-1 generated the strongest signal in the protein samples diluted 1:10 and 1:100. The reactions of ToMV commercial antibodies were different, but weaker. One commercial antibody was undetectable at a dilution of 1:100. Only the AB-1 antibody was capable of detecting ToMV in the protein sample diluted at 1:1000. At the highest used dilution of the protein sample (1:10,000), none of the antibodies were able to detect the presence of ToMV (Figure 2).

**Figure 3:**
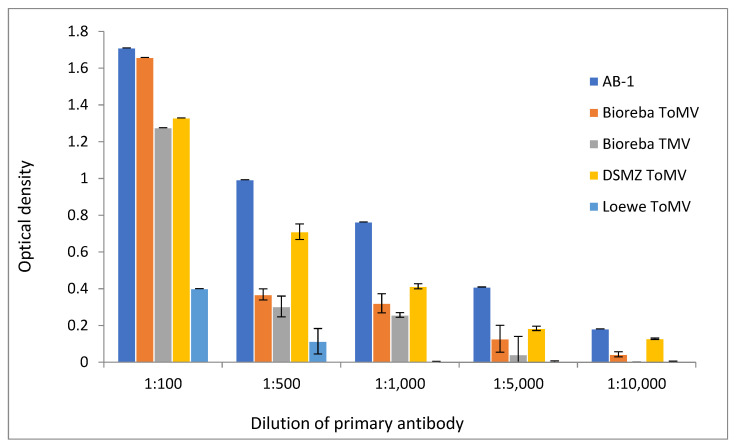
Sensitivity of the differently diluted compared antibodies in ToMV-infected tomato plants by the ELISA assay using five antibodies (new-developed AB-1, three commercial against ToMV—Bioreba ToMV, DSMZ ToMV, Loewe ToMV, and one commercial against TMV—Bioreba TMV) (mean values and standard deviation error bars).

Different dilutions of all used primary antibodies were also tested by ELISA. The concentrations of all antibodies used in analyses, commercial as well as AB-1, were adjusted to the same value of 5 µg/µL of total proteins. The sensitivity at antibody dilutions of 1:100; 1:500, 1:1000; 1:5000; and 1:10,000 (*v*/*v*) was the strongest in the newly developed AB-1 antibody (Figure 3). Figure 3 demonstrates the result after 10 min of incubation. However, almost exactly the same results were obtained after the other incubation periods (10 s, 1 min, and 30 min). The AB-1 antibody showed higher sensitivity in the detection of ToMV than commercially available analogs. A comparison of the antibodies’ sensitivity at dilutions of 1:1000 and 1:5000 showed statistically significant higher sensitivity (*p* < 0.05) of the AB-1 antibody against all others in both dilutions.

**Figure 4:**
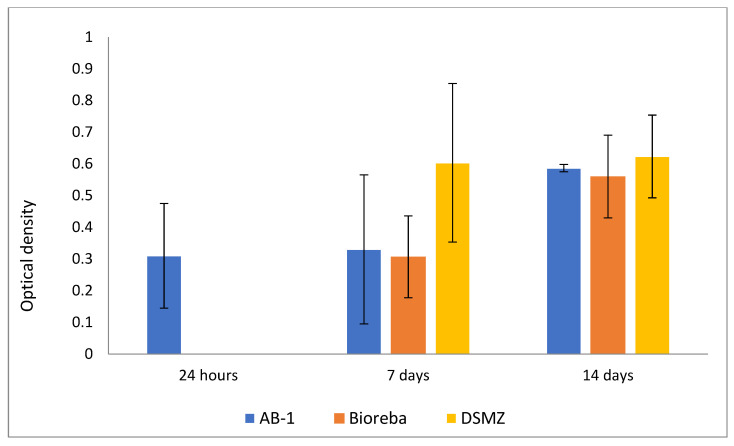
ELISA detection of ToMV at different times after plant inoculation with virus using three antibodies (new-developed AB-1, two commercial—Bioreba, DSMZ) (mean values and standard deviation error bars).

The AB-1 antibody and two commercial antibodies (Bioreba and DSMZ) were tested by ELISA for the ability to detect the virus in ToMV-positive plants of the Niki Zel F1 variety as soon as possible after virus infection. A different leaf from the plant than the one infected with the virus was always used for analysis. Only the AB-1 antibody was able to confirm the presence of ToMV very early on, only 24 h after artificial infection. Late infection was tested one and two weeks after inoculation, and all antibodies were able to detect the virus. Figure 4 summarizes the results of this testing at different times after the infected plants.

### 3.3. Specificity and Cross-Reactivity of AB-1 Antibody

**Figure 5:**
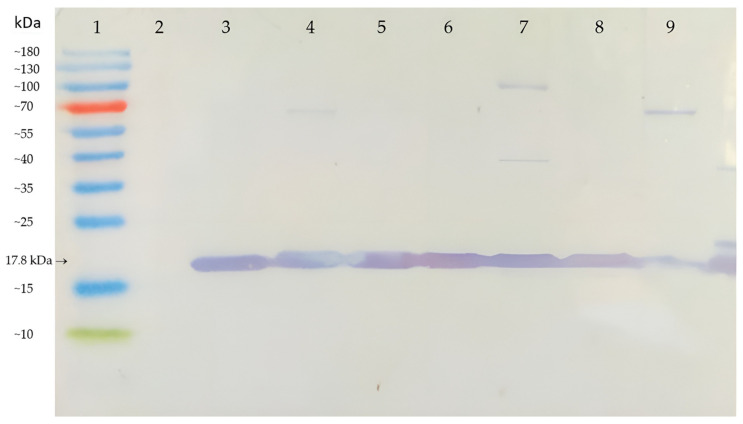
Reactivity of AB-1 antibody with ToMV isolates, TMV, and PMMoV using Western immunoblotting analysis. Lanes: 1—protein ladder, 2—negative control, 3—ToMV PV-0141, 4—ToMV PV-0143, 5—ToMV PV-0846, 6—ToMV PV-1180, 7—ToMV PV-0135, 8—ToMV SL-1, 9—PMMoV PV-0166, and 10—TMV PV-0107.

The basic parameter of any antibody is the ability to detect all isolates of the given virus, but at the same time, not to cross-react with other, albeit very similar, viruses. Altogether, six ToMV isolates (SL-1, PV-0141, PV-0143, PV-0846, PV-1180, and PV-0135), one TMV isolate (PV-0107), and one PMMoV isolate (PV-0166) were used for the artificial infection of tomato plants (variety Niki Zel F1). Plants were scored for systematic infection by Western immunoblotting analysis using the AB-1 antibody. All ToMV isolates as well as TMV and PMMoV reacted with the AB-1 antibody (Figure 5). The size of the detected protein fragment (17.8 kDa) corresponded to the expected size of the coat proteins of TMV, ToMV, and PMMoV, according to data from the UniProt database [31].

**Figure 6:**
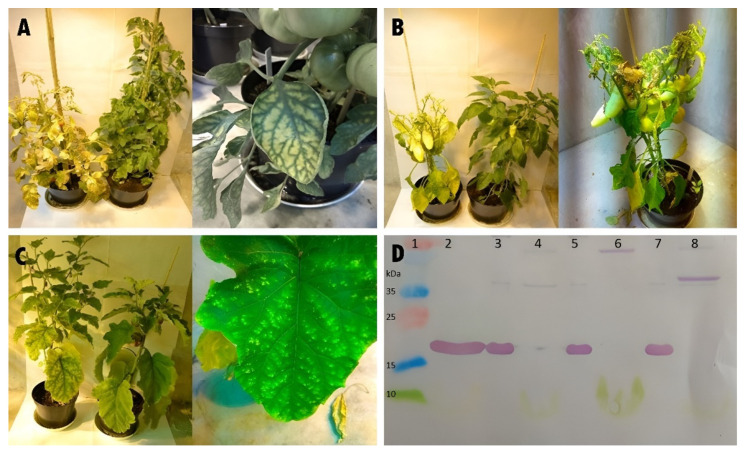
Symptoms of the ToMV SL-1 isolate in tomato, pepper, and eggplant. (**A**)—symptomatic infected tomato plant (**left** in pair) and control plant (**right** in pair), and leaf mosaic symptoms (**right**), (**B**)—symptomatic infected pepper plant (**left** in pair), control plant (**right** in pair), and leaf shedding from infected plant (**right**), (**C**)—symptomatic infected eggplant plant (**left** in pair), control plant (**right** in pair), and mild chlorosis on leaf (**right**), (**D**)—Western immunoblot detection of ToMV in plants of tomato, pepper, and eggplant. Lanes: 1—protein ladder, 2—positive control (virus), 3—infected tomato, 4—negative control tomato, 5—infected pepper, 6—negative control pepper, 7—infected eggplant, and 8—negative control eggplant.

The cross-reactivity of the AB-1 antibody was tested in pepper and eggplant. The pathological symptoms that occurred demonstrated the aggressiveness of the ToMV isolate SL-1 in all three plant species. The infected plants exhibited stunted growth and curling and mottling of the leaves. Symptoms began to manifest as a slight veining of the leaves, which led to a typical mosaic of green and yellow chlorotic spots that subsequently led to necrosis. The fruits of the infected tomatoes ripened later than those on the healthy control plants, were smaller, and had a variety of symptoms, including uneven coloration, a roughened fruit surface, and brown necrotic spots. The symptoms gradually led to the death of the infected tomato plants. The infected pepper and eggplant plants also showed symptoms associated with the presence of ToMV, such as leaf mosaic on eggplant and a hypersensitive reaction inducing necrotic lesions on the pepper plant, followed by the discoloration and shedding of leaves (Figure 6A–C). Western immunoblotting analysis confirmed the positive reactions of the AB-1 antibody with extracts from ToMV SL-1-infected plants of tomato, pepper, and eggplant. These reactions unambiguously detected the protein fragment that matched the size of 17.8 kDa (Figure 6D).

### 3.4. AB-1 Antibody in ToMV Analysis in Roots and Seeds

**Figure 7:**
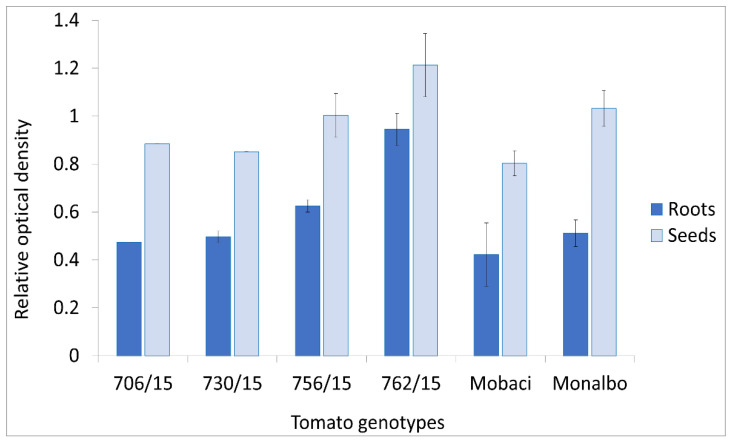
Detection of ToMV SL-1 in roots and seeds of infected tomatoes using the AB-1 antibody (mean values and standard deviation error bars).

Two tomato varieties and four breeding lines infected by ToMV SL-1 were tested by Western immunoblotting for the presence of the virus in roots and seeds. The presence of ToMV was confirmed both in seeds and roots of all infected tomato genotypes (Figure 7). The AB-1 antibody also proved its functionality in these samples. The lowest accumulation of ToMV in seeds as well as in roots was detected in the tomato variety Mobaci, declared as a tobamovirus-resistant genotype through the *Tm-1* resistance gene [36]. Breeding lines 762/15 and 756/15 generated higher reaction signals in the roots and line 762/15 generated higher reaction signals in the seeds than the Monalbo variety declared as the susceptible variety to ToMV infection. Generally, the ToMV SL-1 isolate infected all tested tomato genotypes to a large extent. Thus, the AB-1 antibody can also be used to detect the presence of ToMV in tomato seeds produced for seed trading. From the transmission point of view, statistically significantly higher virus accumulation was observed in the seeds compared with the roots (*p* < 0.05). Among the genotypes, tomato line 762/15 accumulated a statistically significantly higher level of virus than all of the others (*p* < 0.05).

### 3.5. AB-1 Antibody in Analysis of ToMV Persistence and Resilience

**Figure 8:**
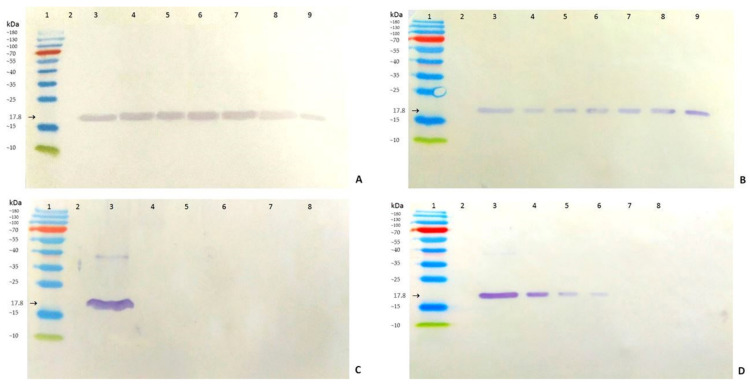
Persistence and resilience of ToMV in soil rhizosphere and root residues analyzed by Western immunoblots 24 months after the removal of the infected tomato plants from the soil. (**A**)—rhizosphere sample, (**B**)—root residues. Lanes: 1—protein ladder, 2—negative control, 3—positive control (ToMV SL-1), 4—line 706/15, 5—line 730/15, 6—line 756/15, 7—line 762/15, 8—Mobaci variety, and 9—Monalbo variety. (**C**)—Same rhizosphere samples after sterilization (tomato line 706/15), (**D**)— same root residues after sterilization (tomato line 706/15). Lanes: 1—protein ladder, 2—negative control, 3—positive control (before sterilization), 4—sterilization for 1 min, 5—sterilization for 2 min, 6—sterilization for 5 min, 7—sterilization for 10 min, and 8—sterilization for 20 min.

*Tobamoviruses* are known to be persistent genetic elements that are able to survive long-term in the environment where they previously infected plants [10]. The virus can remain infectious for a long time in residues from these plants. Therefore, the presence of the ToMV SL-1 isolate was detected in soil rhizosphere samples and in root residues that remained in the soil where infected tomato plants were grown previously. The soil and root samples collected and analyzed 24 months after removing the infected plants from the soil still contained a detectable amount of ToMV. ToMV SL-1 was confirmed by Western immunoblot analysis in all samples (17.8 kDa protein band marked in Figure 8A) from all six tomato genotypes cultivated in the soil previously. The immunochemical reactions from the soil and root residue samples were as high as those in the positive control (Figure 8A,B). *Tobamoviruses* also have extremely stable virions [37]. The resilience of ToMV evaluated in the same soil and root residues after sterilization confirmed this trait. A temperature of 120 °C degraded the ToMV coat protein in the soil rhizosphere, even after 1 min of exposition (Figure 8C). The virus in root residues was more resilient. Even after 5 min or 10 min at 120 °C, the ToMV coat protein (17.8 kDa) was detected by Western immunoblotting (Figure 8D).

## 4. Discussion

Antigens for specific antibody development can be prepared by the purification of native virus particles isolated from an infected plant, subsequent cloning of a relevant gene from the viral genome (most commonly encoding the coat protein of the virus), immunization of an animal, and activation of the immune response, and by the purification of a desired antibody. However, this process is tedious and time-consuming. The simplified process is the in silico approach, where initial experiments are omitted and antibody production in an animal is induced using information from databases of viral genome and protein sequences, synthesizing of the protein for animal immunization, and subsequent purification of the antibody. Another approach, suitable mainly for the commercial production of antibodies, is the production of recombinant antibodies by the expression of the relevant gene from the virus in the host organism. These three main approaches produce monoclonal, polyclonal, or recombinant antibodies [38].

Monoclonal antibodies for ToMV detection are being developed with the aim to eliminate cross-reactivity with other tobamoviruses invading tomatoes [39]. However, even these may not always be able to completely eliminate cross-reactivity with other *Tobamoviruses*, e.g., with Tobacco Mosaic Virus (TMV). However, most of the developed mono-clonal antibodies for ToMV detection did not cross-react with TMV [40].

Polyclonal antibodies are easier to develop and apply for fast and routine diagnostics in phytopathology and agricultural practice by ELISA and Western immunoblotting detection assays. They recognize multiple similar epitopes of an antigen of interest, resulting in better sensitivity of routine assays. Their polyclonality allows the binding of multiple antigenic determinants of the target, better sensitivity in certain assays, and greater stability [41]. Generally, they seem to be robust as they can potentially detect other similar viruses, despite the fact that their specificity is sometimes lower and potentially false-positive results may be obtained. From the practical application point of view, it is extremely important to detect viruses present in plants, even if there have been small changes in the viral genome that can prevent their detection by more specific methods [19]. Serological tests, such as ELISA, are well-suited for the primary mass screening of virus presence in plants [11,42], and more precise detection methods with satisfactory sensitivity and specificity are reasonable to use after the initial screening [19].

Commercially available ToMV antibodies with an unknown recognition site origin are widely used in virological studies and in agricultural practice. The reason for the development of a new polyclonal antibody for ToMV detection within this study resulted from the absence of detailed information about the recognition site of commercially available polyclonal antibodies. Data regarding the epitope design were not available for commercially available antibodies and thus could not be compared. A common problem during the development of plant virus antibodies is acquiring purified protein for the immunization of laboratory animals [43]. The synthesis of the proposed epitope succeeded in immunizing two rabbits without the tedious process of virus purification. Variation in sera properties between animals, or even between bleedings of the same animal, is considered as an important limitation of polyclonal antibody production [26]. The immunization of two rabbits by the epitope designed in this study was conducted to check possible biological bias and immunochemical response variation of the antibody based on the characteristics of individual rabbits used for the production of the antibody [44]. However, no significant difference between the antibodies produced by the two different rabbits was noted (results not presented).

Newly developed polyclonal antibodies must have similar or better diagnostic properties, detection limit, and desired cross-reactivity than those of commercial ones. The antibody presented in this work was developed by designing an epitope-based peptide antigen (coat protein) of a specific ToMV isolate, SL-1. The high functionality of the developed polyclonal antibody (AB-1) and detection properties comparable to or better than those of commercially available analogs were confirmed by Western immunoblotting and ELISA analyses. The AB-1 antibody generated a stronger signal in extracts from the ToMV-infected plant in comparison with commercially available antibodies. It was the only antibody capable of detecting virus particles in the plant sap diluted at the ratio of 1:1000, which indicated its detection sensitivity. At the highest used dilution of antibody (1:10,000), the AB-1 antibody generated the strongest signal. It should be repeated here that the sensitivity of all antibodies compared (AB-1 and commercial) was tested at the same protein concentrations in the antibodies.

In addition, AB-1 was the only one of the tested commercial antibodies for detection that was capable to detect ToMV in the very early phase of plant infection, as early as 24 h after virus inoculation. This may be a result of the epitope design aiming for a high antigenic response [32,33]. Throughout this study, the AB-1 antibody was able to reliably detect ToMV in different vegetative and generative organs (leaves, roots, and seeds) of the infected tomato and could be considered as suitable for large-scale plant sample screening. The AB-1 antibody also detected ToMV persisting in the rhizosphere soil and root residues two years after plant cultivation and also after short-term sterilization at a high temperature.

In addition to tomato plants, the AB-1 antibody was also capable of clearly detecting the presence of ToMV SL-1 in artificially infected pepper and eggplant plants. Polyclonal antibody AB-1 proved to be capable of detecting all available ToMV isolates and another two tested tobamoviruses (TMV and PMMoV). The cross-reactivity of the ToMV antibody with PMMoV observed here was not reported for the commercial antibodies tested in this study. Generally, the sensitivity and other parameters of non-commercial polyclonal antibodies developed by researchers for their specific applications vary. For example, antibodies developed for the detection of Pepper yellow leaf curl Thailand virus (PepYLCTHV) showed positive cross-reaction with Tomato leaf curl New Delhi virus (ToLCNDV), both belonging to the genus *Begomovirus* [45]. Polyclonal antibodies developed for Potato virus Y (PVY) and Potato leaf roll virus (PLRV) using synthetic peptides were highly sensitive and detected both viruses in infected samples better than those from commercial ELISA kits. In contrast, the antibody produced against Potato virus X (PVX), although it provided positive detection, had a weaker signal than that obtained by the antibody from the commercial kit [46]. The same procedure in the development of polyclonal antibodies for immunodiagnostic detection was used for Sugarcane mosaic virus (SCMV) [47] and other viruses. They sometimes had better parameters than commercially available antibodies for the same viruses, sometimes worse. Polyclonal antibodies were also developed non-commercially for the detection of *Tobamoviruses*. The antibodies developed for Pepper mild mottle virus (PMMoV) specifically detected another four *Tobamoviruses* (including ToMV) in an antibody dilution of 1:1000 and infected plant sap dilution of 1:5120 [48]. The polyclonal antibody developed for the detection of ToMV exhibited twice as high sensitivity in ELISA testing as commercial antibodies [49]. The titer of the antiserum was 1:1024 and the dilution end point of purified ToMV was 1:32.

The polyclonal antibody, AB-1, developed in this study is suitable for use in agricultural practice and research. Its parameters are the same or better than those of commercial ToMV antibodies. This antibody, by immunochemical detection techniques (ELISA and Western immunoblotting), can also be applied in the wide screening of ToMV in tomato germplasm (e.g., in plant genetic resource collections), parental genotypes and breeding lines (tomato breeding programs), and in tomato seeds during the seed certification process, i.e., in activities where the number of samples is high [50]. Simple and routine immunochemical testing, mainly ELISA, is also appropriate in the detection of viruses, virus spreading, and plant–virus interactions at the agro-ecological interface [51], which were related to the development of an antibody against ToMV in our experiments within this study. Sequences of modified oligopeptides with antigenic potential for the immunization of animals and production of antibodies are released for a wide range of diagnostic applications within this study.

## Figures and Tables

**Table 1:** Sequences of primers used for the amplification of ToMV SL-1 (bp—base pair).

Primer (Amplicon Length)	Forward Primer	Reverse Primer
CP1 (128 bp)	5′-CAGATTTCCTGGCGATGTTT-3′	5′-TCGGACTCTGCTGGTTTTCT-3′
CP2 (439 bp)	5′-TGGTCATCTGTATGGGCTGA-3′	5′-AAGATGCAGGTGCAGAGGTC-3′
P1 (867 bp)	5′-CACAAACAGCCACATCGTCC-3′	5′-ACCTCTCTATTAGAGGCTGG-3′
P2 (1361 bp)	5′-CGAGAGGGGCAACAAACATG-3′	5′-GTAAGACCACTCTCGTTGCT-3′
P3 (1715 bp)	5′-AGGTCTGAGTGGGATGTCGA-3′	5′-ACAGGATTGATAGACGCAGC-3′
P4 (742 bp)	5′-CTTGTTGTGTCCGGTGAGTG-3′	5′-CCACACCTCGCTGAACTGCT-3′
P5 (505 bp)	5′-AGGTCTGAGTGGGATGTCGA-3′	5′-GTAAGACCACTCTCGTTGCT-3′
P6 (707 bp)	5′-ACGTCGCATTACTGGAGCAC-3′	5′-CACAGGATTGATAGACGCAG-3′

## Data Availability

Not applicable.
